# Leveraging Experimental Strategies to Capture Different Dimensions of Microbial Interactions

**DOI:** 10.3389/fmicb.2021.700752

**Published:** 2021-09-27

**Authors:** Gunjan Gupta, Amadou Ndiaye, Marie Filteau

**Affiliations:** ^1^ Département des Sciences des aliments, Université Laval, Québec, QC, Canada; ^2^ Institut sur la Nutrition et les Aliments Fonctionnels (INAF), Québec, QC, Canada; ^3^ Institut de Biologie Intégrative et des Systèmes (IBIS), Université Laval, Québec, QC, Canada

**Keywords:** experimental systems, network biology, phenotype, ecological interactions, functional interactions, systems biology, microbial interaction patterns

## Abstract

Microorganisms are a fundamental part of virtually every ecosystem on earth. Understanding how collectively they interact, assemble, and function as communities has become a prevalent topic both in fundamental and applied research. Owing to multiple advances in technology, answering questions at the microbial system or network level is now within our grasp. To map and characterize microbial interaction networks, numerous computational approaches have been developed; however, experimentally validating microbial interactions is no trivial task. Microbial interactions are context-dependent, and their complex nature can result in an array of outcomes, not only in terms of fitness or growth, but also in other relevant functions and phenotypes. Thus, approaches to experimentally capture microbial interactions involve a combination of culture methods and phenotypic or functional characterization methods. Here, through our perspective of food microbiologists, we highlight the breadth of innovative and promising experimental strategies for their potential to capture the different dimensions of microbial interactions and their high-throughput application to answer the question; are microbial interaction patterns or network architecture similar along different contextual scales? We further discuss the experimental approaches used to build various types of networks and study their architecture in the context of cell biology and how they translate at the level of microbial ecosystem.

## Introduction

Microorganisms form complex communities that perform essential functions in all ecosystems, impacting plants, animals, and humans (Antoniewicz, 2020). Interactions within microbial communities may be crucial to these ecosystems’ functions, if for example a function depends on the presence of complementary species (Hansen et al., 2007). In fact, interactions may sometimes be as important as the presence of an individual species (Gould et al., 2018). Therefore, understanding microbial communities at the system level, that is considering interactions between microorganisms, is a major challenge of fundamental importance (Abram, 2015; Blasche et al., 2017; Otwell et al., 2018). In the twentieth century, interest in microbial interactions has been primarily focused on inhibitory interactions because of the applications of antimicrobials in the field of medicine. Emphasis on interactions with indirect mechanisms (*via* inhibitory metabolite production) formed the traditional experimental approach (Zhang and Straight, 2019). Since the beginning of the twenty-first century, our interest in microbial interactions has grown toward elucidating fundamental ecological principles to better understand microbial communities and their behavior (Gorter et al., 2020). At the same time, technologies enabling high-throughput experiments are becoming available, offering opportunities to characterize biological systems on an unprecedented scale and depth (Hsu et al., 2019; Kehe et al., 2019, 2020; Sanchez-Gorostiaga et al., 2019; Temkin et al., 2019; Ma et al., 2020).

**Figure 1:**
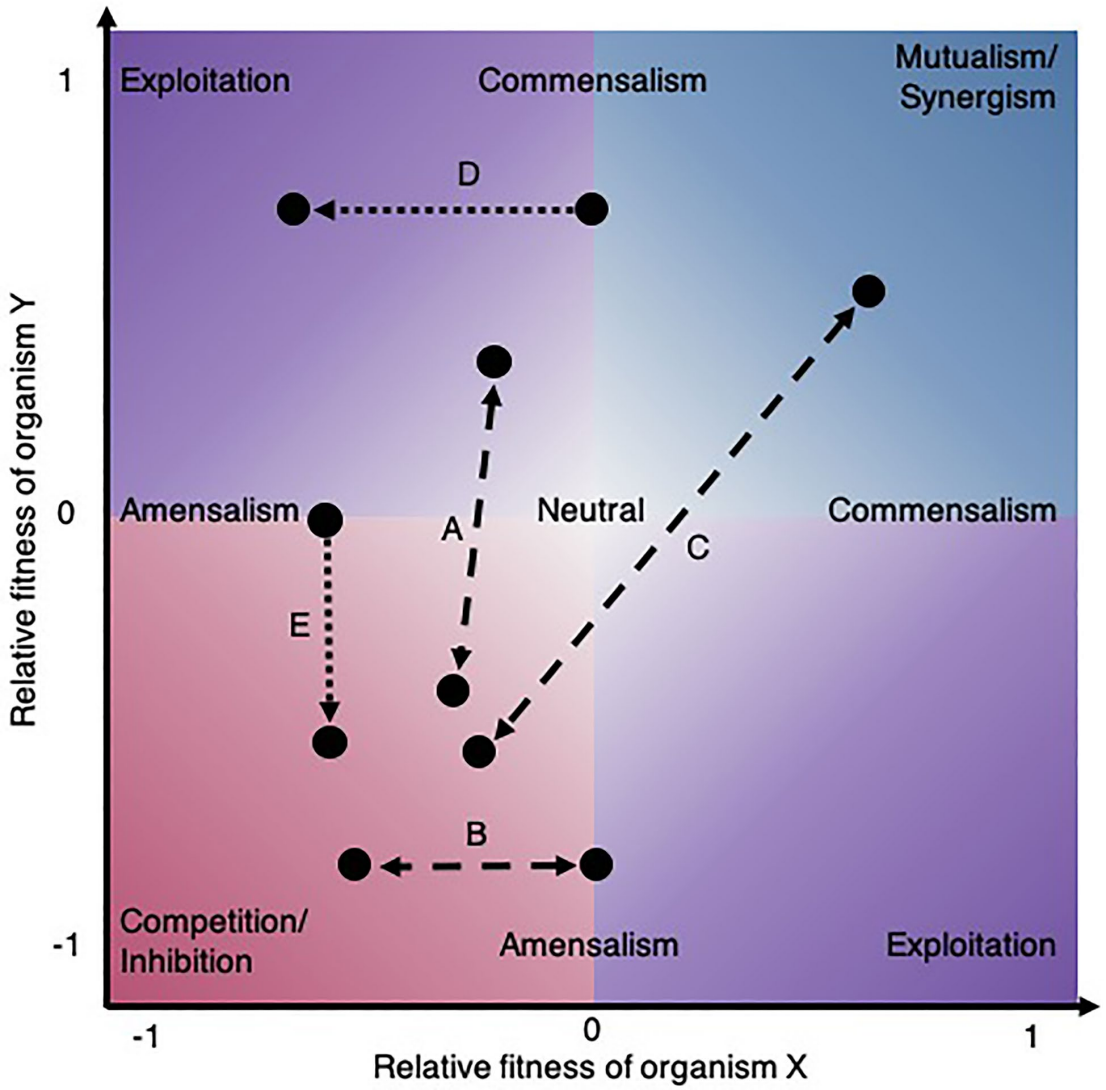
Shift in microbial ecological interactions. The environmental context can change the ecological interactions between species (dashed lines). **(A)** For instance, the interaction between lung bacteria was shown to switch from exploitation to competition in response to a carcinogenic compound (Liu et al., 2017). **(B)** Changes in nutrients alter ecological interaction outcomes as demonstrated for *Acinetobacter johnsonii* and *Pseudomonas putida*, fluctuating between amensalism and competition depending on the carbon source provided (Rodríguez-Verdugo et al., 2019) or **(C)** by the ammonia concentration switching cooperation to competition between an alga and a fungus (Zuñiga et al., 2019). Evolution also leads to shifts in ecological interaction between species (dotted lines). **(D)** For example, a commensal interaction between two bacteria evolved into exploitation in a two-species biofilm (Hansen et al., 2007) and **(E)** an amensal interaction between a yeast and a bacterium rapidly evolved into inhibition (Andrade-Dominguez et al., 2014).

A unidirectional microbial interaction can be regarded as the net effect of an organism on another over a given period and can be measured by quantifying phenotypic differences in the presence and absence of the partner strain (Hsu et al., 2019). From an ecological perspective, considering the bidirectional effects on fitness or growth of both partners (Figure 1), these relationships can be classified as exploitative (exploitation, predation, parasitism), cooperative (synergism, mutualism), competitive (inhibition), one-sided (commensalism, amensalism), or neutral (Pacheco and Segrè, 2019). However, this static framework is limited because it does not reflect the dynamic and context-dependent nature of interactions (Figures 1A–E) and the net outcome may result from a trade-off of multiple molecular strategies at once (Pacheco and Segrè, 2019). For example, pairs of bacteria can simultaneously compete for one substrate and mutually cross-feed other molecules (D’Hoe et al., 2018). Moreover, from an applied perspective, microbial fitness may not be the most relevant phenotype to study, but rather activity (Pishchany et al., 2018), production (Islam et al., 2020; Senne de Oliveira Lino et al., 2021), behavior (e.g., coaggregation, virulence or biofilm formation; Diaz and Valm, 2020), morphology (Cheong et al., 2021), or benefit to the host (Gould et al., 2018). Also, the nature of microbial interactions may be ecological when the effect results from population dynamics or it may be behavioral when partners alter each other’s phenotype, or a combination of both (Sanchez-Gorostiaga et al., 2019). Therefore, depending on the perspective, different approaches considering various phenotypic traits may be appropriate to characterize microbial interactions. From a mathematical standpoint, interactions are the effects of variables that are not simply additive (or linear) and thus can be measured on a variety of quantitative traits given the appropriate experimental design and null model.

Bottom-up approaches that try to predict community behavior by modeling a small number of microbial interactions are appealing because they can offer great mechanistic insight into various interaction types and properties (Rodríguez-Verdugo et al., 2019; Liu et al., 2020a). However, because these approaches are based on a few microorganisms and conditions, they do not capture the high dimensionality of microbial interactions imparted by their complex and diverse nature. Altogether, the different interaction types, strengths, and contexts (time, space, community size, environmental gradient, host, etc.) may form various microbial interaction patterns that, in turn, form distinct network topologies. To map and understand the architecture of these networks, microbial interactions need to be characterized at high density by testing a large proportion of possible interactions in each community and context. To achieve this end, systematic approaches, i.e., system biology approaches that can reflect a wide diversity of relationships are needed. Moreover, there is a need to experimentally evaluate predicted interactions in microbial interaction network studies (Lv et al., 2019). Approaches to experimentally capture microbial interactions are essentially combinations of culture methods and phenotypic characterization methods. In this review, through our perspective of food microbiologists, we present and discuss diverse approaches and their ability to measure different dimensions of ecological and functional microbial interactions, and further map microbial interactions and networks. We then discuss how experimental approaches used to build various types of networks in the context of cell biology translate to microbial ecology.

## Capturing Microbial Interactions While Accounting for Their Complex Nature

The relevance of studying microbial interactions has been revealed over the last decade along with our understanding of microbial community functions across all habitats on Earth (Braga et al., 2016; Tshikantwa et al., 2018). With the growing interest among researchers from different fields of science and the growing number of microbial interaction-based applications (Imam et al., 2017; Tshikantwa et al., 2018), the complexity of interactions between microorganisms is being unraveled. The ability to understand the complex nature of microbial interactions in the most fundamental way requires knowledge of their attributes, for instance, their mode of action and the factors that influence these interactions. In the following section, we first discuss the attributes of microbial interactions relevant to experimental approaches to map ecological interactions, then factors intrinsic and extrinsic to microbial communities influencing microbial interactions.

**Figure 2:**
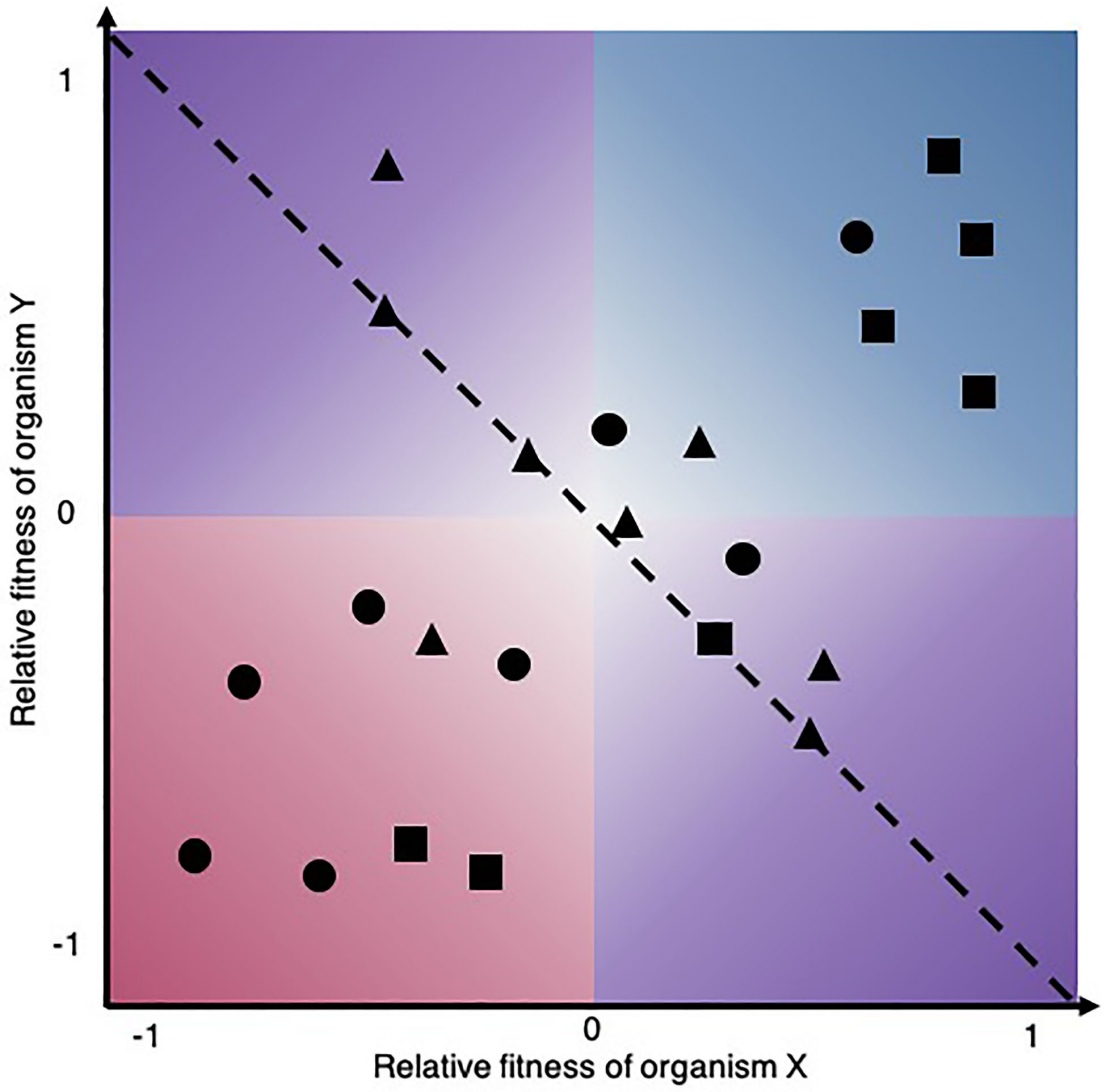
Different microbial ecological interaction patterns may be revealed by distinct experimental approaches. Shapes in the biplot symbolize hypothetical microbial interactions mapped by different approaches among the same set of microorganisms that may reveal interactions mediated by contact-dependent mechanisms, diffusible compounds, or volatile compounds for instance. The dashed line partitions interactions where the net effect on community growth is positive (upper right) and negative (lower left), while they are neutral along the line.

The complexity and multidimensionality of microbial interactions are a conundrum that has already been acknowledged (Pacheco and Segrè, 2019). For our purpose, we define the attributes of a microbial interaction as the inherent properties of the relationship that constitute variables (categorical or continuous) that can be used to describe them. We focus on attributes that are of fundamental importance for experimental approaches able to map ecological interactions, that is, to place pairwise interactions on the bidimensional space of Figure 2. Thus, the first attribute to consider in this context is the reciprocity of microbial interaction assays, as some approaches are unidirectional, meaning that they only measure the effect of one microorganism on the other, while other are bidirectional (Table 1). In the case of unidirectional assays, the experiment must be performed twice to obtain the reciprocal information needed to map the outcome on a Cartesian plane. The directionality itself is the qualitative outcome, i.e., the sign of the effect of one microorganism on another. Microbes can have net negative, neutral, or positive effect on one another and sometimes qualitative information of directionality may be sufficient to categorize interactions into relationship types and observe patterns (Yan et al., 2021). However, the strength, that is the quantitative magnitude of these effects, can vary and have important implications for the biodiversity and stability of microbial communities (Ratzke et al., 2020). Moreover, considering the strength of the reciprocal relative effect on fitness reveals the more complex nuance of certain types of ecological interactions. When placing interactions on a Cartesian plane, it becomes obvious that commensalism and amensalism occupy only a linear trajectory where the relative fitness of one member is constrained to zero, whereas the other types of ecological interactions can be asymmetric, i.e., the strength of the relative effect on fitness is not equal (|X|≠|Y|). Thus, the distribution patterns of microbial relationships, even for a given ecological interaction type, can vary, which may have important consequences for microbial communities. Also, strength is a critical attribute to consider for microbial interaction mapping since weak interactions require a higher number of replicates to achieve statistical significance (Hsu et al., 2019). Since strength is an attribute required to map microbial interactions, all approaches reported in Table 1 are quantitative.

**Table 1:** Quantitative approaches to study ecological interactions between microorganisms and their ability to capture different microbial interaction attributes.

Approach			Attributes captured					References
Culture system	Method to measure fitness/growth	Throughput^1^	Bidirectional	Contact dependent	Volatile Compounds	Feedback	Dynamics	
Growth in conditioned media	Optical density	Medium	No	No	No	No	No	Biggs et al., 2017; Ponomarova et al., 2017; Gu et al., 2020b; Ratzke et al., 2020; Blasche et al., 2021
Liquid co-cultures separated by membranes		Low	Yes	No	No^2^	Yes	Yes	Moutinho et al., 2017; Jo et al., 2021
Liquid co-culture assay	qPCR with specific primers	Low/medium	Yes	Yes	No	Yes	No	Medlock et al., 2018; Weiss et al., 2021
	Amplicon sequencing combined with optical density.	Medium	Yes	Yes	No^2^	Yes	Yes	Venturelli et al., 2018
	Amplicon sequencing with spiked-in standard	Medium	Yes	Yes	No^2^	Yes	No	Blasche et al., 2021
Sandwich agar culture plates	Plate counts	Medium	No	No	Yes	Yes	No	Cosetta et al., 2020
Stamped colonies on agar	Colony area	Low	Yes	No	Yes	Yes	No	Liu et al., 2017
Automated spot-on lawn co-culture assay		Medium	No	Yes	No^2^	Yes	No	Blasche et al., 2021
Glass Petri dish microcosms	Optical density and plate counts	Low	Yes	No	Yes	Yes	No	Garbeva et al., 2014
Droplet printing with defined micron-scale patterning.	Quantification of fluorescently labelled cells using fluorescence microscopy.	Low	Yes	Yes	Yes	Yes	Yes	Kumar et al., 2021
Microfluidic droplets.		High	Yes	Yes	No	Yes	Yes	Hsu et al., 2019
Microfluidic device.		Low	Yes	No	No	Yes	Yes	Gupta et al., 2020
Microwell recovery arrays		High	No	Yes	No	Yes	Yes	Barua et al., 2021
kChip: droplets within microwells		High	Yes	Yes	No	Yes	Yes	Kehe et al., 2020

1 Assay format: low=<96, medium=96–500, high=500+.

2 Volatile compounds produced in assays on the same plate may cause interference in these approaches.

Another important attribute of microbial interactions is their mode of action. Interactions can act *via* direct or targeted mechanisms such as antimicrobials (Tyc et al., 2014), quorum quenching (Grandclément et al., 2016), or indirect mechanisms such as siderophore (Gu et al., 2020a), EPS, or acid production (Ratzke and Gore, 2018). Interactions between microorganisms are also bound to be mediated by cell surface molecules such as antigens, fimbriae, flagella, pili, and exopolysaccharides. These structures can affect colony morphology, population boundaries, chirality, and migration patterns, which in turn can influence interactions between competing colonies (Jauffred et al., 2017). The various microbial interaction modes of action were extensively reviewed by Braga et al. (2016). Depending on the underlying mechanism, interactions can be classified as contact-dependent or contact-independent. Contact-dependent interactions require cells to come in physical contact (Konovalova and Sogaard-Andersen, 2011), whereas contact-independent interactions can act over a distance (Phelan et al., 2012). The distance can be short when they are mediated by soluble compounds or long when mediated by volatile compounds (Schmidt et al., 2015; Schulz-Bohm et al., 2015; Tyc et al., 2015). Also, some effects may only be captured if feedback between microorganisms is possible, as for example the production of costly secondary metabolites may not be constitutively expressed and may require an induction signal (Pacheco and Segrè, 2019; Zhang and Straight, 2019). In a natural context, multiple mechanisms may mediate an interaction between two microorganisms. However, a given experimental setup may only capture part of these effects. Dedicated approaches have been applied to reveal specific modes of action, for instance, volatile compounds or siderophore-mediated interactions (Cosetta et al., 2020; Gu et al., 2020b). It would be interesting to compare the resulting microbial interaction patterns as shown in (Figure 2) for the same set of microorganisms using approaches capturing complementary interaction types.

Finally, biological interactions are highly dynamic, but they are often reported as a single snapshot. Per their definition, microbial interactions are effects measured over a defined period. Thus, time is also an attribute of microbial interactions. An important consideration about time is that this attribute may co-vary with contextual factors even in controlled laboratory conditions, as microorganisms can alter their environment. For example, Ratzke and Gore (2018) reported a change in the type of interaction between microorganism over time because of changes in pH. From an experimental perspective, the choice of period is often arbitrary and varies between approaches and studies. An ideal way to capture the temporal variation of microbial interactions would be to measure real-time responses (Moutinho et al., 2017; Gupta et al., 2020) which is not always experimentally feasible without disturbing the system or may come at the cost of detecting only specific types of interactions (Table 1). As interspecies interactions may occur over different time scales, hours, days, or weeks (Rivett et al., 2016; Venturelli et al., 2018), care must be taken while designing an approach to capture microbial interactions. Indeed, microbial interaction dynamics on the long timescale can be related to the evolution of interspecies interactions as cell populations in long co-cultures can be subjected to natural selection and evolution (Goers et al., 2014). Gorter et al. (2020) have reviewed the evidence showing that the evolution of interspecies interactions is an important driver of microbial community properties and dynamics. Another aspect of studying temporal variations in microbial interactions is the order of colonization of isolates which is an element of historical contingency (Fukami, 2015). For instance, in the context of surface biofilms, it was shown that pre-colonization of a surface by a single species could initially modulate the ability of other strains to adhere to that surface later (Lapointe et al., 2019). Similarly, in the context of a host, priority effects can influence community structure (Martínez et al., 2018) and preexisting endogenous microbiota composition can influence the colonization of exogenous species (Maldonado-Gómez et al., 2016). Moreover, the temporal component of microbial interactions may be affected by positive or feedback loops in the ecosystem, such as in the case of a host response (Lozupone et al., 2012). Therefore, understanding the temporal dynamics of microbial interactions is crucial for studying the assembly and (in)stability of microbial communities in various habitats.

## Experimental Approaches to Measure Ecological Interactions

Measuring microbial interactions requires the development and use of experimental approaches combining culturing techniques with phenotypic assays. In Table 1, we report recent studies that used various culture-dependent methods to explore ecological interactions between microorganisms and compare their ability to capture different microbial interaction attributes. Co-culturing microorganism *in vitro* is the most common experimental setup to measure and characterize interactions between microorganisms. However, it is technically possible to map microbial interactions without co-culturing microorganisms by performing reciprocal classical conditioned media assays. Typically, in these assays, one microorganism is cultured in the cell-free supernatant, i.e., spent media of another microorganism. For example, Biggs et al. (2017) performed a spent-media screen to elucidate the potential for interactions and metabolic capabilities of a model microbial community, Ponomarova et al. (2017) performed a conditioned media assay to identify the yeast secretome components that enable the growth of LABs, Ratzke et al. (2020) performed a spent media experiment to test the influence of nutrient concentration on interaction strength between soil bacteria, and Gu et al. (2020b) used treated spent media assays to measure siderophore-mediated effects. This approach has the advantage that time and space components of the interaction can be uncoupled (Blasche et al., 2021); however, it cannot capture contact-dependent interactions or feedback effects. As many microorganisms depend upon metabolite production by fellow community members for survival (Ponomarova et al., 2017), this approach is helpful mainly to study these kinds of cross-feeding interactions. Other approaches involve some form of co-culture systems, which have been reviewed elsewhere (Bogdanowicz and Lu, 2013; Goers et al., 2014; Nai and Meyer, 2018; Tan et al., 2019). Briefly, these systems allow microorganisms to be co-cultured on solid plates (Liu et al., 2017), in liquid media (Kheir et al., 2018), or on a submerged surface (Lapointe et al., 2019). Microbes can be perfectly mixed (Smid and Lacroix, 2013) or spatially separated (Goldschmidt et al., 2021), depending on the experimental setup. Another emergent experimental setup for measuring and characterizing microbial interactions is microfluidic cell culture systems (reviewed in Burmeister and Grunberger, 2020). These systems can allow to co-culture microorganisms with full spatio-temporal resolution at high-throughput (Hsu et al., 2019; Kehe et al., 2020; Barua et al., 2021). However, these methods have been coupled with fluorescence-based detection methods which limit their throughput application for mapping binary interactions in diverse organisms. Indeed, to map ecological interactions, a fitness or growth measurement for each partner is required. Methods to measure the impact of microbial interactions on growth or fitness require either knowledge of the absolute abundance of each member of the community separately, or relative abundance of each member multiplied by the absolute abundance of the community. In the first approach, a quantitatively measurable trait unique to each member is required, to measure each member abundance separately, e.g., colony morphology (Traxler and Kolter, 2015), growth on selective media (Cheong et al., 2021), fluorescent tag (Conacher et al., 2020), specific enzymatic activity (Rivett et al., 2016; Liu et al., 2020a), specific DNA sequence (Kumar et al., 2019; Blasche et al., 2021), or a physical separation allowing for individual quantification (Liu et al., 2017; Moutinho et al., 2017; Jo et al., 2021). Physical separation is useful to study uncharacterized isolates; however, it precludes the detection of contact-dependent interactions. In the second approach, a relative abundance estimate can be obtained by microbial community profiling methods such as high-throughput sequencing (Pishchany et al., 2018; Blasche et al., 2021), combined to a compound measure of absolute abundance [Optical density (Venturelli et al., 2018), quantitative polymerase chain reaction (qPCR; Medlock et al., 2018; Weiss et al., 2021), total plate counts (Cosetta et al., 2020), etc.] or a spiked-in quantification standard (Blasche et al., 2021). This second approach can also be used when considering phenotypes other than fitness. Taken together, current approaches all present a trade-off between throughput and some attributes captured. Moreover, while they are promising approaches, high throughput methods developed so far require custom equipment and are limited by the availability of fluorescently tagged strain.

## Contextual Gradients Affecting Microbial Interactions

**Figure 3:**
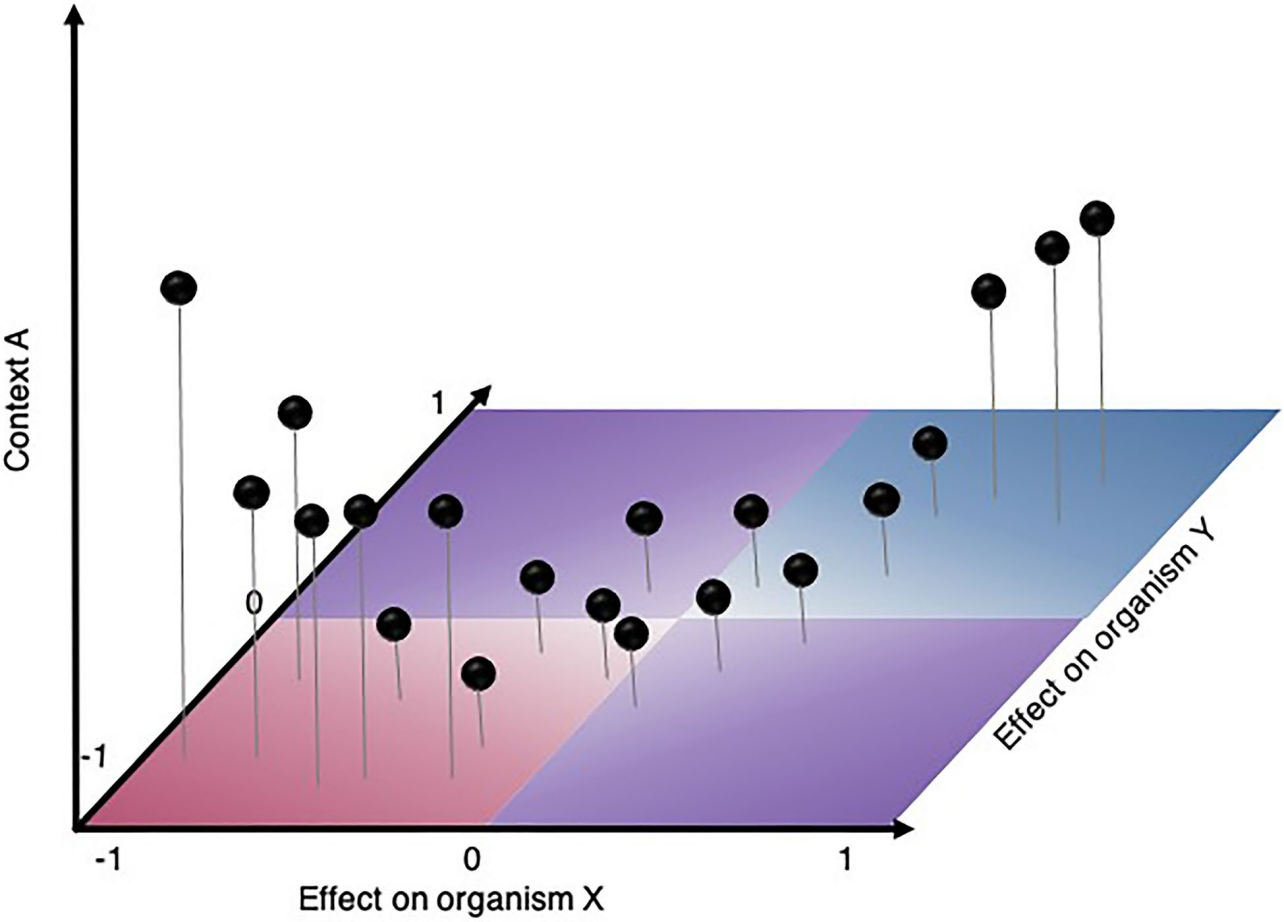
Hypothetical scenario illustrating how experimentally mapping binary interactions at high throughput on a range of phenotypes (X- and Y-axis) and in various contexts (Z-axis) will help answer the outstanding question in microbial ecology: are there common patterns driving microbial relationship? For instance, do interactions strengthen in specific contextual gradients? Experimental approaches needed to answer such questions may vary according to the contextual gradient (e.g., time, space, level of structure, phylogenetic gradient, environmental gradient, community diversity, etc.).

Answers to some outstanding questions in microbial ecology can be given by mapping microbial interactions in various contexts, e.g., time, space, phylogenetic gradient, environmental gradient, community structure, community diversity, etc. (Figure 3). These contextual gradients affecting microbial interactions are factors that can be viewed as either intrinsic or extrinsic properties of the microbial system. Understanding how microbial interaction distribution vary along these factors may ultimately help understand driving forces that shape microbial relationships.

Microbial communities vary in their species diversity, structure, size, and density, which can influence the interactions between microbes within that community. Hence, these properties of microbial communities are intrinsic factors affecting microbial interactions. First, community diversity is a quantitative measure of both the variety or number of different microorganisms (species richness) and their relative distribution (evenness; Xu et al., 2020). How the diversity of microorganisms living in a community affect community functions is an important question in ecology that has been explored (Zha et al., 2016; Purswani et al., 2017; Yu et al., 2019). For example, Zha et al. (2016) found that higher initial diversity of natural freshwater bacterioplankton communities promoted higher levels of richness and evenness in local communities which ultimately affected the functional performance of communities. Though community diversity plays a critical role in interactions between microorganisms, only a few experimental studies addressed the effect of community diversity on microbial interaction patterns (Rivett et al., 2016). Second, community structure relates to the composition and the proportion of each member in the community. Microbial ecologists have defined community composition as “who is there” (Nemergut et al., 2014; Harzevili and Hiligsmann, 2017). This factor is important to consider while studying microbial interactions as their outcome may be determined by the composition of the background communities and higher-order interactions. While some microbial interactions are strain dependent as exemplified by genetically engineered consortia of *Lactococcus lactis* strains (Kong et al., 2018), others can be conserved at higher phylogenetic levels (Garbeva et al., 2014; Cosetta et al., 2020). Thus, different levels of microbial interactions may be considered at different phylogenetic scales. Moreover, population structure has a great impact on the evolution of cooperative interactions (Celiker and Gore, 2013). A third intrinsic factor modulating microbial interactions is the population size of the community and how these members are distributed in the community space per unit area or volume, i.e., their density. Experimental methods used to study microbial interactions have a definite environment with a finite space that can be populated. However, very little is known about the effect of change in community size or density on microbial interactions patterns because this variable changes over the course of experiments. However, it is clear from studies on quorum sensing or quorum quenching that this factor can influence microbial interactions (Abisado et al., 2018; Liu et al., 2020b). Overall, it can be said that experimental evidence for the effect of community properties on microbial interaction at the system level is currently scarce. Nevertheless, computational approaches predicting interactions in microbial communities identified responses to perturbations in community properties (Zuñiga et al., 2017). Considering the extensive diversity of microbial life, we have still much to learn about microbial communities and how their intrinsic properties shape the interaction networks within.

The context in which community exists, e.g., space and environmental gradients affect microbial interactions extrinsically. First, how microorganisms are assembled in space has important consequences in their interactions and community functions (Jeckel and Drescher, 2020). Nadell et al. (2016) discussed how spatial arrangement of microbes within a community influences the cooperative and competitive cell–cell interactions. Microbes can interact in a spatially structured or unstructured (well mixed) environment. Spatial arrangement of microbes can lead to specific kinds of interaction in the community (Kumar et al., 2021) and reciprocally, interactions can lead to spatial dispersion or segregation of microbes (Datta et al., 2016). Usually, spatial segregation of microbes in biofilms increases the frequency of interactions between cells of the same genotype and favor cooperative behaviors whereas in a well-mixed environment competition is predominant (Nadell et al., 2016). However, these are not hard rules, and more experimental and theoretical work is being conducted to better refine the link between spatial arrangement of microorganisms and interactions between them. In experimental biology, some of the studies to measure microbial interactions consider spatial configuration in their experimental design (Connell et al., 2013; Peaudecerf et al., 2018; Co et al., 2020; Kumar et al., 2021) and allow us to investigate the link between arrangement of microorganisms in space and ecological outcomes. Alternatively, multiple methods can be combined to assess the effect of structure on ecological interactions (Blasche et al., 2021).

Another important extrinsic factor that mediates interactions between microbes is their environment as microbial communities live in ever-changing conditions (Rodríguez-Verdugo et al., 2019). Environmental context in terms of nutrient availability, pH, temperature, pressure, water activity, water flow, light, etc., can modulate ecological interactions between species (Figure 1). Aside from these abiotic factors, the environmental context of a microbial community can also include biotic factors such as the immune system of a host. Usually, the effect of environmental factors on microbial interactions is experimentally studied by measuring the response of microbes (in terms of growth and other phenotypes) in co-culture experiments. For example, Lin et al. (2016) studied the effect of temperature on microbial interactions among microbes involved in biogas digestion and Tecon et al. (2018) studied the effect of hydration conditions on bacterial interactions among soil isolates in a controlled environment. However, the study of how the environment alters microbial interactions is not always straightforward, because there is a feedback loop between microbes mediating their environment and in turn the change in environment mediating the presence, absence, and the type of interaction among microbes (Khan et al., 2018). Ratzke and Gore (2018) demonstrated experimentally that microbes modify the pH of their environment which feed backs on them and their interactions with other microbes. Although environmental context is such an important factor mediating microbial interactions, there are still many conditions to explore. An approach that captured and compared microbial interactions among a set of 20 different soil bacteria across 40 environments with varying carbon sources was recently introduced (Kehe et al., 2020). Ultimately, such high-throughput experimental approaches will contribute to our understanding of how microbial interactions vary along environmental gradients. Moreover, *in vitro* culture systems enabling the study of model microbiota within the context of a host have been developed such as the HuMiX (Shah et al., 2016), “gut-on-a-chip” system (Kim et al., 2016), and others reviewed by Vrancken et al. (2019). Such systems offer promising avenues to investigate microbial interactions while accounting for biotic factors, as exemplified in Maurer et al. (2019), although so far, their application to map microbial interactions has been limited because most of the studies focused on the impact of host–microbe interactions.

With the increased development and use of high-throughput technologies, understanding of microbial communities and interactions among its members is rapidly transforming. However, some biases in experimental methods are hard to avoid. For example, culture methods used to map interactions have biases toward studying specific type of mode of action which make them well suited only for specific context. Such experimental biases provide different and somehow incomplete information about microbial interactions that may cast a shadow on the actual whole picture of ecological interactions and their effect on the community. In comparison with culture-dependent experimental techniques, *in situ* techniques which capture microbes in their native environment offer a more holistic approach to capture microbial interactions. Examples of recent *in situ* approaches to study microbial interactions include transparent microcosms in a native-like setting that allow visualization and long-term observation of microbes individually and their cell–cell interactions in three dimensions over time (Shank, 2018) or mesocosm experiments to study complex communities and the effect of secondary metabolites on microbial community structure (Patin et al., 2017). Apart from biases in experimental methods, intrinsic and extrinsic factors are often uncontrolled variables in experimental settings. Thus, it is important to explicitly report the context in which microbial interaction are detected. Also, these factors are not entirely independent from each other. For instance, spatial and temporal dynamics are both relevant for microbial interactions mediated by diffusible molecules (Gupta et al., 2020). Therefore, methods to measure the impact of these factors on microbial interactions need to be carefully controlled for these effects. To sum up, studies measuring microbial interactions should clearly report on the various attributes, intrinsic and extrinsic factors mentioned above, as these have all been shown to influence ecological interactions.

## Measuring the Functional Dimension of Microbial Interactions

In some contexts, the impact of microbial interactions on community functions may be much more important than the ecological outcome and therefore knowledge of the absolute abundance of each interacting partner may not be needed. Sometimes even, microbial interactions that affect population size may not alter specific function of the overall community, as was observed for amylase production (Sanchez-Gorostiaga et al., 2019). In Table 2, we report experimental strategies exploiting various kinds of phenotypes including morphology, molecular phenotype, activity or production, social behavior, and impact on host to study microbial interactions. These phenotypes could each be used to map reciprocal interaction effects (Figure 3) to reveal functional microbial interaction patterns. In this section, we discuss how these various phenotypic assays at different levels, from single cells to entire community can be leveraged to capture the functional dimension of microbial interactions.

**Table 2:** Approaches to measure functional microbial interactions.

Phenotype category	Phenotype	Measurement level	Phenotyping method	Organism tracing method	Throughput^1^	References
Morphology	Cellular morphology	Single cell	Flow cytometry	Physical separation by a membrane	Low	Heyse et al., 2019
	Colony morphology	Population	Volatile compounds bioassay	Physical distance		Low	Tyc et al., 2015
Molecular phenotype	Cellular fingerprints	Single cell	Raman spectroscopy	Physical separation by a membrane	Low	Heyse et al., 2019
	Cell surface molecular profile	Population	Imaging mass spectrometry	Physical distance	Low	Chen et al., 2020b
	Metabolomic profile	Community	Nuclear magnetic resonance spectroscopy	Binary combination	Low	Medlock et al., 2018
	Gene expression profile	Community	RNA sequencing	Binary combination	Low	Zuñiga et al., 2019; Ikeyama et al., 2020; Strub et al., 2021
Activity or production	Antimicrobial activity	Community	Agar plug diffusion	Physical separation by a permeable membrane	Low	Pishchany et al., 2018
		Population	Spot on lawn	Binary combination	Low	Weiss et al., 2021
	Respiration	Community	CO_2_ indicator	Random partition combinatorial design, flow cytometry and T-RFLP	Low	Rivett et al., 2016
	Enzymatic activity	Community	Enzymatic assays	Combinatorial assembly	Low	Sanchez-Gorostiaga et al., 2019
	Ethanol yield	Community	HPLC	Flow cytometry and combinatorial assembly	Medium	Senne de Oliveira Lino et al., 2021
	Acidification	Community	Colorimetric assay with pH indicator in the culture media	Binary combination	Medium	Blasche et al., 2021
Social behavior	Coaggregation	Community	Visual coaggregation assays	Binary combination	Medium	Kumar et al., 2019
	Biofilm	Community	Biofilms formation assay in bioreactor	Combinatorial assembly and selective plate counts	Low	Lapointe et al., 2019
	Dispersal	Population	Distance	Binary combination	Low	Zhang et al., 2018
Impact on host	Gene expression levels	Community	Microarrays and RT-qPCR	Binary combination	Low	Shah et al., 2016
	Intracellular metabolite levels	Community	Metabolomic analysis	Binary combination	Low	
	Reproductive success, longevity and development	Community	Plate counts	Combinatorial assembly	Low	Gould et al., 2018

1 Assay format: low=<96, medium=96–500.

Microorganisms exhibit a vast array of phenotypic traits, some that can be measured in individual cells (García-Timermans et al., 2020) and others that emerge only in the context of a population or community (Grandclément et al., 2016; Diaz and Valm, 2020). Morphology is a phenotype expressed at the level of cells or populations forming colonies. At the cellular level, flow cytometry is suitable for studying cellular features such as size, shape, and surface properties using light scattering and has been used along with a binning grid to measure the effect of microbial interactions (Heyse et al., 2019). However, this technique is limited to a descriptive interpretation as it does not provide information on the mechanistic or functional aspect of the interaction. At the population level, microbial colony morphology may undergo visible changes in response to compounds produced by nearby species. For instance, exposure of *Serratia marcescens* to volatile compounds produced by *Chryseobacterium* sp. or the mixture of *Dyella* sp. and *Janthinobacterium* sp. leads to an increased circularity of *S. marcescens* colonies (Tyc et al., 2015). While in this case, this approach can be used because volatile-mediated interactions are being sought, in the case of mixed communities the inference of microbial interactions from the analysis of colony morphology could be much more complex. For this reason, there is a scarcity of studies on the effect of microbial interactions on colony morphology, even if the latter is a classical phenotype.

Modifications induced by microbial interactions can also occur at the molecular level, which can be revealed by fingerprinting, profiling and omic methods. First, for an in-depth study of microbial interactions at the cellular level, Raman spectroscopy can be very useful for the evaluation of biochemical phenotypes. Indeed, this method presents a complete image resulting from combination of the individual spectra of the different cellular components (nucleic acids, fatty acids, proteins, etc.). The resulting information is specific to each cell, distinguishing it from others, like a unique signature (Lorenz et al., 2017; García-Timermans et al., 2020). This method was used to study the phenotypic heterogeneity of two drinking water isolates, *Enterobacter* sp. and *Pseudomonas* sp. The study found that bacterial interactions can be a modulating factor for phenotypic heterogeneity in mixed cultures (Heyse et al., 2019). Since phenotypic heterogeneity plays an essential role in virulence and drug susceptibility strategies, understanding the impact of microbial interactions at the cellular level may be crucial (Weigel and Dersch, 2018). Second, microbial interactions can also affect cellular metabolism which can be revealed using various metabolomic methods. For instance, applying nuclear magnetic resonance spectroscopy to co-cultures of strains from a model mouse microbiota, *Clostridium* and *Parabacteroides* were shown to increase their utilization of lactose and some amino acids while producing more propionate and other amino acids (Medlock et al., 2018). The spatial distribution of secreted metabolites is also a phenotype that can reveal microbial interactions. Using Matrix-Assisted Laser Desorption/Ionization Time of Flight (MALDI-TOF) Imaging Mass Spectrometry (IMS), a study reported interspecies metabolic interactions with suppressions, increases and exchange of metabolites between *Microcystis aeruginosa* and its antagonist *Pseudomonas grimontii* (Chen et al., 2020b), further describing the molecular mechanisms involved in the previously established ecological interaction between these two species (Sandrin and Demirev, 2018). Finally, microbial interactions can alter gene expression, which can be measured at the genomic level using metatranscriptomics (Zuñiga et al., 2019). For instance, the transcriptome analysis of *Fusarium verticillioides* and a *Streptomyces* strain showing antifungal activity revealed an alteration of the expression of 18,5 and 3,8% of genes upon interaction, respectively (Strub et al., 2021). This asymmetric impact on the transcriptome was also observed on the magnitude of change, which was greater in the mold (Strub et al., 2021). When the genomes of organisms are well annotated, transcriptomic analysis of interacting microbial pairs can be particularly useful to decipher functional interactions such as metabolite exchange. As an example, the investigation of the microbial interaction between *Phascolarctobacterium faecium* and the gut commensal *Bacteroides thetaiotaomicron* using RNA sequencing pointed to the exchange of several metabolites including succinate, vitamin B_12_, and glutamate (Ikeyama et al., 2020). Altogether, omic methods contribute to a better understanding of the mechanisms at play between interacting species, as they allow to systematically measure all molecular phenotypes of a given type at once. However, some phenotypes can be particularly complicated to study depending on the system. Indeed, for transcriptomic and proteomic data, mapping RNA or peptide sequences to closely related (genetically homologous) members in a community can prove difficult and lead to inaccuracies (Melin, 2004; Diz et al., 2012).

A combination of methods can be employed to measure microbial interactions through different phenotypes. For instance, Rivett et al. (2016) tracked respiratory activity to dissect interactions between members. A random partition design was used to set up communities where every species is sampled equally for each level of species richness, then flow cytometry and Terminal Restriction Fragment Length Polymorphism (T-RFLP) were used to assess the abundance and relative proportions of bacterial populations, respectively. They mapped binary interactions between bacteria at several time points and showed a change in microbial interaction patterns, the interactions shifting from antagonist to neutral. This study also revealed that the strength of microbial interactions has an impact on the respiratory activity of the community, the reduction of binary interactions was associated with a decline in respiratory activity (Rivett et al., 2016). Therefore, using functional phenotypes to map microbial interactions can be useful to explore their patterns, but also to understand how they impact microbial community functions.

Studying functional microbial interactions is also relevant from an applied perspective, since they can improve the performance of biotechnological processes by modulating community composition and functionality (Hall et al., 2018). In a consortium with other bacteria and *Saccharomyces cerevisiae* strain PE-2, *Lactobacillus amylovorus* induced a 3% increase in ethanol yield from sugarcane fermentation. In addition, *Lactobacillus amylovorus* established a cross-feeding relationship *via* the production of acetaldehyde which has a positive impact on the growth of *S. cerevisiae* (Senne de Oliveira Lino et al., 2021). The acidification ability of a strain is one of the main criteria in the selection of starters used in dairy products and may also contribute to pathogens inhibition (Reda et al., 2018; Samedi and Linton Charles, 2019). To pinpoint interactions modulating acidification, Blasche et al. (2021) tested and compared monocultures and cocultures of kefir isolates in a media containing bromocresol purple as pH indicator. The results showed positive synergistic interactions on 15 pairs of interactions in comparison to monocultures.

In some cases, the production of microbial compounds of interest is only observed in an interaction situation because of induction effects (Tyc et al., 2014). This was the case for certain actinomycetes that can produce a wide variety of compounds of medical or industrial interest (antibiotics, antifungal compounds, anticancer agents, etc.; Traxler et al., 2013). Indeed, the interaction of *Streptomyces coelicolor* with five other actinomycetes resulted in the production of different compounds specific to each interaction. Microbial communication in coculture can unlock silent biosynthetic gene clusters leading to the production of new metabolites (Rutledge and Challis, 2015). For instance, when interacting with *Tsukamurella pulmonis*, a mycolic acid containing bacterium, *Streptomyces lividans* produces a red pigment. Activation of this cryptic biosynthetic pathway requires contact with living cells and the presence of mycolic acid as a precursor for red pigment production (Onaka et al., 2011). It has also been reported that some silent biosynthetic gene clusters present in *Aspergillus nidulans* are activated in coculture with soil-dwelling actinomycetes (Schroeckh et al., 2009). This exemplifies how production of certain compounds by microorganisms often depends on the interactions that microorganisms have. Therefore, microbial interactions could be an important player in the discovery of new antimicrobials. For instance, Pishchany et al. (2018) discovered amycomicin, an antibiotic produced by *Amycolatopsis* sp. AA4 when interacting with *Streptomyces coelicolor* M145. Amycomicin is a modified fatty acid containing an epoxide isonitrile that acts as a potent inhibitor specifically targeting *Staphylococcus aureus* and was shown to reduced infection in a mouse model (Pishchany et al., 2018). In another study, *Streptomyces endus* S-522 in combination with *Tsukamurella pulmonis* produced Alchivemycin A, a new antibiotic that specifically targets *Micrococcus luteus* (Onaka et al., 2011).

Microbial interactions can not only impact metabolite production, but they can also alter social behaviors, such as biofilm formation or coaggregation, which are important for the survival, proliferation, resistance, and pathogenicity of microorganisms (Kumar et al., 2017). Coaggregation is defined as a cellular mechanism of adhesion with a very specific recognition between genetically different microorganisms (Afonso et al., 2021). There are several advantages in coaggregation for bacteria such as increased virulence and resistance, more efficient metabolism, exchange of genetic information and chemical signals (Afonso et al., 2021). A study on 27 human skin bacteria showed 123 combinations with positive coaggregation. *Staphylococcus haemolyticus*, *Acinetobacter* spp. and *Pseudomonas* spp. formed more positive coaggregations than the others. These species could be involved in microbial transmission by hand due to their high potential to coaggregate (Kumar et al., 2019).

Another widespread social behavior of microorganisms is biofilm formation, a major problem in many fields such as medicine or the food industry (Verderosa et al., 2019). Phenotypic changes may also occur to ensure better growth and survival in biofilms (Hendrickson et al., 2017; Mutha et al., 2019; Afonso et al., 2021). Biofilms are associated with a higher pathogenicity, but also with negative effects on the organoleptic quality of food products due to the secretion of lipolytic or proteolytic enzymes and on their persistence industrial infrastructures (Galie et al., 2018). Biofilms can also show a certain resistance to sanitation (Fagerlund et al., 2016). The economic losses related to biofilms are not negligible (Galie et al., 2018). *Leuconostoc pseudomesenteroides*, *Lactobacillus plantarum*, and *Pseudomonas fluorescens*, common spoilage bacteria in the meat industry showed synergistic interactions when growing in a mixed biofilm culture compared to monocultures (Lapointe et al., 2019). Furthermore, pre-colonization of a surface by a single species initially could modulate the ability of other strains to adhere to that surface later (Lapointe et al., 2019).

The aptitude of bacteria to disperse in a matrix may depend on biotic and abiotic parameters, including microbial interactions. Dispersal is used by some bacteria to conquer more resource-rich spaces, but this motility may depend on microbial interactions and the latter may ultimately modulate the community composition. A study conducted on a cheese rind microbial community showed that motile bacteria used the physical structures of filamentous fungi for dispersal (Zhang et al., 2018).

Finally, the functional impact of microbial interactions may also extend to effects on a host but owing to the complexity and diversity of these systems, our understanding remains limited (Hassani et al., 2018; Figueiredo and Kramer, 2020). Still, experimental models of reduced complexity can be used to highlight functional microbial interactions on host traits. Using the HuMix *in vitro* microfluidic model, the impact of the interaction between *Lactobacillus rhamnosus* GG and a *Bacteroides caccae* strain was measured on epithelial intestinal cell gene expression and intracellular metabolite levels (Shah et al., 2016). In *Drosophila*, interactions between the five major gut bacteria have shown a relationship between these interactions, microbiome abundance and host lifespan, but development and fecundity did not appear to depend on microbial interactions (Gould et al., 2018).

Through these few examples, it is apparent that microbial interactions are complex and can be expressed by phenotypes other than growth and fitness. Functional interaction phenotypes offer new perspectives for bioprocess performance (Hall et al., 2018; Senne de Oliveira Lino et al., 2021), in pathogen control (Pishchany et al., 2018) and in agriculture (Woo and Pepe, 2018). Several methods or approaches can be employed to investigate microbial interactions depending on the target phenotype. These methods may be classical such as antimicrobial activity and enzymatic assays or more advanced such as omics. To properly map functional microbial interactions, microorganism tracing methods are required to capture the bidirectional effects. As reported in Table 2, binary combinations and physical separation appear as popular methods to study functional microbial interactions. However, the number of functional microbial interactions studied with these methods in a single study is very limited. To further explore functional microbial interaction patterns with these methods, a higher throughput needs to be achieved. High-throughput methods, though complex in some cases, could be an important asset for unraveling the role of interactions in specific microbial community functions and thus contribute to applications development in several fields.

## Scaling Up Systems Biology Approaches From Cellular Systems to Microbial Ecosystems

**Figure 4:**
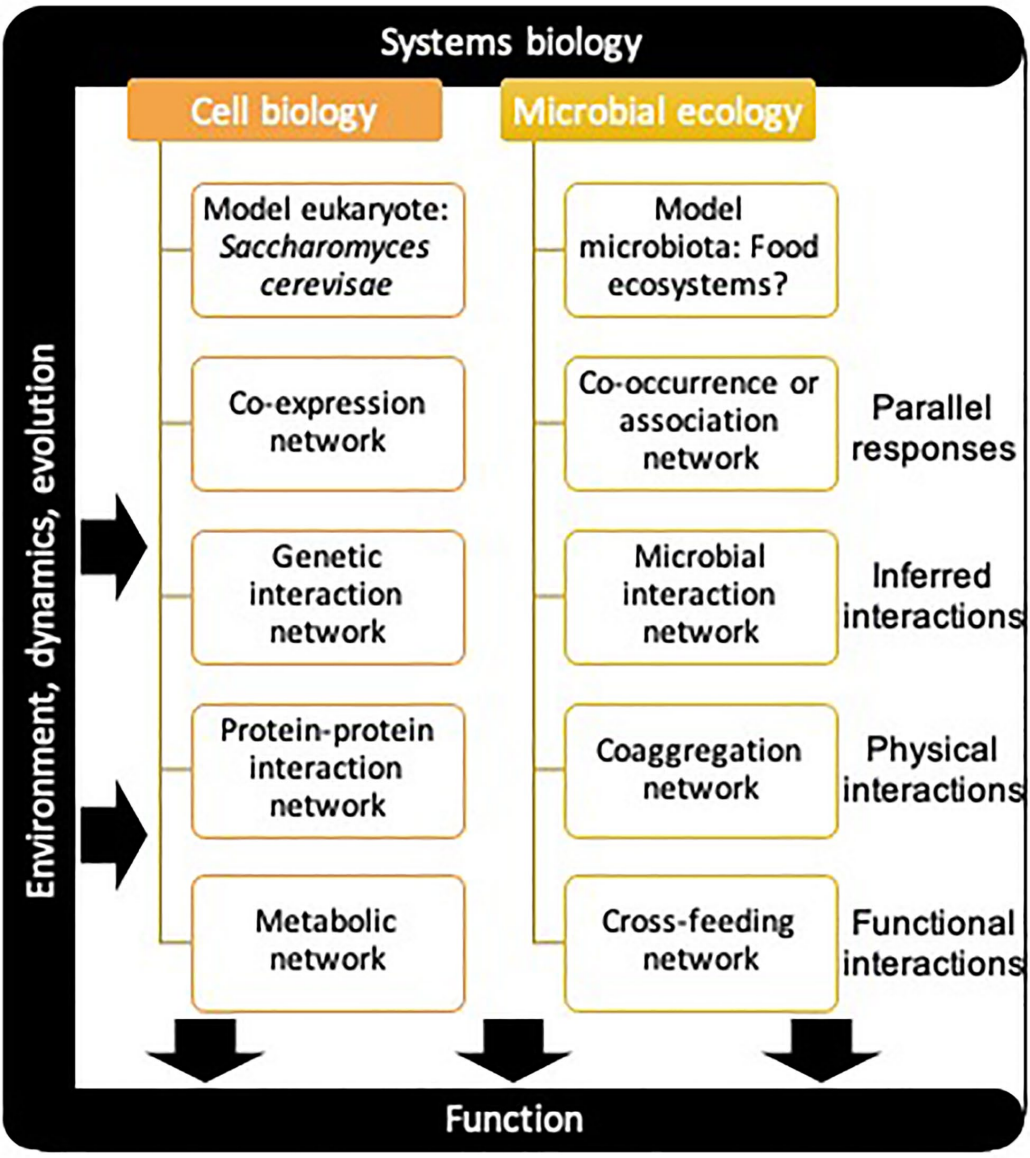
Parallel between systems biology approaches in cell biology and microbial ecology. Different relationship types can be considered between the systems components at both the cellular and community level to make up various networks.

The study of microbial communities has evolved rapidly since the advent of high-throughput sequencing. The challenge of observing phylogenetic diversity patterns is now mostly resolved, but we are still a long way from dissecting the assembly, organization, and functions of multi-species microbial communities (Wolfe and Dutton, 2015). Indeed, despite the accumulating terabytes of omic datasets, the information about microbial interactions within communities is not readily accessed (Abram, 2015) and the mechanisms driving diversity pattern and assembly of microbial communities remain modestly described (Nemergut et al., 2013; Gralka et al., 2020). This fundamental problematic falls within the scope of systems biology, that aims to understand how the components of a system interact and work together. Since its advent at the turn of the century, systems biology has had a tremendous impact on the field of cell biology and human medicine (Chuang et al., 2010), but microbial ecology is now beginning to adopt and apply these conceptual advances (Abram, 2015; Blasche et al., 2017; Otwell et al., 2018). So far, we reviewed approaches that address the challenge of identifying and measuring ecological and functional interactions within microbial communities. The next key challenge is to accurately represent them in theoretical frameworks (Widder et al., 2016), such as networks or landscapes. The application of network theory to complex biological systems can prove useful in identifying complex and emergent patterns and to help fill the gap between structure and function at different scales (Gosak et al., 2018). Much of the work that has been done in that respect at the cellular scale could be conceptually transferred to the microbial community scale (Figure 4). In the following discussion, we outline examples of cell biology networks and link them to their conceptual equivalent in microbial ecology.

Microbial communities are complex systems which can be defined as “a system whose collective behavior is difficult to derive from a knowledge of the system’s components” (Lv et al., 2019). The inherent complexity of natural microbial communities makes it difficult to test most hypothesis directly. Model systems of reduced complexity where each variable can be controlled provide a way to test ecological theories (Heyse et al., 2019; Bengtsson-Palme, 2020). Indeed, cell systems biology benefited greatly from the choice of a simple but powerful model, the budding yeast *Saccharomyces cerevisiae*, a eukaryotic cell with relatively few components (~6,000 genes), easily grown and genetically manipulated in laboratory conditions (Giaever and Nislow, 2014; Marsit et al., 2017), and for which tractable strains collections were created and made available to the scientific community (Giaever and Nislow, 2014). To achieve the same goal in systems microbiology, one would need to select a simple model ecosystem, avoiding pathogens, bypassing the need for a host, and that could be easily grown and manipulated in laboratory settings. Microbial model ecosystems and synthetic microbial communities have been around for decades (Jessup et al., 2004) but started getting momentum about 10years ago (according to a keyword search in Pubmed) and have been the subject of reviews and opinions in fundamental and applied research since (De Roy et al., 2014; Großkopf and Soyer, 2014; Blasche et al., 2017; Bengtsson-Palme, 2020; Zaramela et al., 2021). Their usefulness is undisputable, yet the context-dependent nature of microbial interactions begs the question; is there an ideal model? Probably not since each applied field will eventually need its own model system, but microbial ecology could benefit from a shared model to identify unifying principles. Again, getting inspiration from cell biology and their model *S. cerevisiae*, an important organism to produce wine, beer, and bread, a food-derived microbial community could fit the requirements of a simple model ecosystem. Indeed, some authors have successfully used phylogenetically diverse fermented food ecosystems as models (Wolfe and Dutton, 2015; Cosetta and Wolfe, 2020; Blasche et al., 2021), while others have designed synthetic communities of engineered lactic acid bacteria (Kong et al., 2018; Liu et al., 2020a). A clear advantage of food microbial communities is that they have been well characterized with respect to several abiotic gradients because of their importance for food production and preservation. Ultimately, whatever the choice of model, concerted efforts to construct and share tractable collections of microorganisms, such as barcoded and/or fluorescently labeled strains would provide a valuable common resource to the scientific community. Laboratories with different expertise could then collaborate more easily to map microbial interactions with complementary methods. Moreover, barcoded strains could offer the possibility of tracking phylogenetically close strains that cannot be distinguished by phylogenetic biomarkers such as 16S rDNA and thus fill this gap on the phylogenetic scale.

Using a suitable model system, a simple type of relationship between the elements of that system can be derived from the observation of their parallel response across conditions. In cell biology, this principle was applied to gene expression in *S. cerevisiae* to construct a gene co-expression network (van Noort et al., 2004). In this context, the approach has proven useful to understand the nature of transcription regulation. In microbiology, an increasing number of descriptive metabarcoding and metagenomic datasets are becoming available and these can be used to infer parallel microbial relationships. Different mathematical approaches can be used for network inference and microbial community modeling, their challenges and applications have been reviewed in (Dohlman and Shen, 2019; Matchado et al., 2021; Zaramela et al., 2021). Multiple cross-sectional and longitudinal studies used co-occurrence or co-abundance to generate association networks from a variety of environments (e.g., Faust et al., 2015; Ke et al., 2019; Zotta et al., 2019; Chen et al., 2020a; Harrison et al., 2020; Moran-Ramos et al., 2020). A meta-network encompassing samples at the earth scale has even been constructed (Ma et al., 2020). Such networks allow to identify groups of microorganisms that follow similar occurrence or relative abundance patterns. When these networks are created from descriptive data, the nature of the predicted relationships cannot be resolved, as co-occurring microorganisms may simply respond to the same environmental cues and not affect each other. Their usefulness is therefore limited in some respects and their pitfalls have been debated elsewhere (Faust and Raes, 2012; Dohlman and Shen, 2019; Lv et al., 2019), but recently developed methods incorporating metadata and absolute count data may help improve their accuracy (Tackmann et al., 2019; Yoon et al., 2019) as well as construction from experimental data gathered in controlled conditions (McClure et al., 2020). Moreover, integration of other annotations such as meta(genomic) annotations can be included to improve functional interpretation (Röttjers and Faust, 2018). Microbial association networks are to date the largest networks depicting microbial relationships and allow to study their architecture on an unprecedented scale.

Relationship from the elements of a system can also be implicitly inferred using an appropriate experimental design. In cell biology, when studying genetic interactions, i.e., genetic epistasis, genes are removed to create simple, double, or multiple knockout organisms. Nearly all binary genetic interactions have been mapped in *S. cerevisiae* (Costanzo et al., 2016) and thousands of higher-order interactions from triple mutants have been reported (Kuzmin et al., 2018). Importantly, in this approach, removing elements from the system allows to infer their effect in the context of all other elements present, which cannot be evaluated while performing binary co-cultures (Venturelli et al., 2018; Blasche et al., 2021). Indeed, testing binary interactions may reveal only part of the true relationships taking place in the context of a community (Wolfe et al., 2014; Zhang et al., 2018; Gralka et al., 2020). Therefore, approaches that systematically consider the community context are needed to fully address these fundamental questions. The strategy of constructing single knockout (or leave one out) community has been applied on small microbial consortia (Kato et al., 2005; Gutiérrez and Garrido, 2019). In microbial ecology, the strategy can even be extended to include full combinatorial assembly, which is not possible with genes because a minimum number of genes are required for cell viability. So far, the strategy has been exploited in small consortium of a few members to address how species interactions and higher-order interactions shape ecosystem diversity and function (Gould et al., 2018; Sanchez-Gorostiaga et al., 2019; Senne de Oliveira Lino et al., 2021). Using combinatorial microbial assembly, Gould et al. (2018) measured the effect of microbial interactions between the five core species of the fly *Drosophila melanogaster* on the host fitness by adapting the mathematics of genetic epistasis. Similarly, borrowing theory from fitness landscape, Sanchez-Gorostiaga et al. (2019) were able to disentangle binary and higher-order interactions in a consortium of six starch-degrading soil bacteria, effectively mapping its functional landscape. Notably, they used amylolytic activity as the function of interest to infer microbial interactions, with and without population dynamics. Industrial ethanol fermentation by *S. cerevisiae* is another model for which the microbial interactions between contaminating bacteria have been dissected to map its functional landscape (Senne de Oliveira Lino et al., 2021). In this context, they demonstrated that although competitive bacterial interactions are common, higher-order interactions buffer their negative effects on ethanol yields. While these studies comprehensively studying interactions in small model consortium have underlined the importance of higher-order interactions, the alternate approach of extensively mapping binary interactions may still prove useful to scale up our system understanding, particularly if this can be accomplished in a community context. Full combinatorial assembly is a laborious endeavor for complex microbial communities because the number of interactions to test increases exponentially with the number of species (Gould et al., 2018). Instead, constructing only simple and double knockout microbial consortium would allow to infer binary microbial interactions in the context of the community, which may be feasible at higher throughput. One of the greatest challenges to overcome is how to handle community assembly at high throughput, at a speed fast enough to match microbial growth. Technologies such as kChip (Kehe et al., 2019, 2020), microwell recovery arrays (Barua et al., 2021) and microfluidic droplets (Hsu et al., 2019) are proving promising in that respect, but still have limitations such as small population sizes and the need for strain labeling. The construction of the network based on genetic interactions profiles in *S. cerevisiae* (Costanzo et al., 2016; Kuzmin et al., 2018) was possible in part because of automated colony manipulations technologies, some of which are adaptable to study microbial interactions (Blasche et al., 2021), which could prove useful to the field of microbial ecology in the future.

Protein–protein interaction networks report physical interactions between molecular components of the cell. In *S. cerevisiae*, several methods have been employed to map binary interactions and the resulting networks have helped understand protein complexes and the organization of the cellular interactome (Uetz et al., 2000; Tarassov et al., 2008; Yu et al., 2008). Physical contacts between molecular components also occur outside cells. In microorganisms, coaggregation reflect the physical attachment or adherence between genetically distinct cells which is highly specific (Katharios-Lanwermeyer et al., 2014; Kumar et al., 2019). This type of interaction is of particular relevance in oral biofilm (Diaz and Valm, 2020), but literature on the topic outside of that field has been scarce despite its ubiquity (Katharios-Lanwermeyer et al., 2014; Stevens et al., 2015). A notable example is a study on human skin isolates where the network of coaggregation is reported for 27 bacteria (Kumar et al., 2019). Besides coaggregation, other types of contact-dependent interactions exist in the microbial world such as “T6SS dueling” where antibacterial effector proteins are translocated *via* the bacterial type VI Secretion System (T6SS) in heterologous species (Basler et al., 2013). Another example of interaction between microorganisms outside the typical ecological scope that sometimes involve physical contact is horizontal gene transfer. The transfer of genetic material between organisms can occur indirectly *via* transformation or transduction and directly *via* conjugation. Notable, a network of horizontal gene transfer was reported for the human gut microbiota (Li et al., 2020). These examples illustrate that mapping contact-dependent interactions in general will help account for a wider diversity of relationships and may help uncover new mechanisms of interaction.

Cellular metabolic network of *S. cerevisiae* (Forster et al., 2003; Heavner et al., 2012) represent biochemical reactions, connecting metabolites with gene products, providing a mechanistic understanding of interacting components of the system. A simple equivalent is constructing a bipartite network connecting metabolites with microbial species using or producing them as was performed by (Venturelli et al., 2018). Their study showed that resource utilization and phylogeny are not necessarily coupled, highlighting the need to understand functional relationships between microbes such as trophic interactions, which are believed to be central drivers of microbial community assembly (Gralka et al., 2020). A consumer resource model explicitly accounting for cross-feeding at the microbial community scale was developed and then compared to microbial community data from the Earth microbiome project (Marsland et al., 2019). Genome-scale metabolic modeling and community scale flux simulations have also been used to systematically explore the impact of resource competition and metabolic cross-feeding on microbial community composition (Machado et al., 2021). However, such model still requires experimental validation that has yet to be performed on the same scale. Thus, experimental approaches combining some form of binary or combinatorial assembly of model communities and metabolomic profiling as the phenotype such as performed in (Medlock et al., 2018), would allow to construct consumer resource networks based on experimentally validated cross-feeding interactions and to test the hypothesis generated by these models.

Another type of functional interaction at the cellular level is represented by the relationship between genotype and phenotype. Screening of various mutant collections helped to map these functional relationships in *S. cerevisiae* and annotate genomic data (Hillenmeyer et al., 2008), although hundreds of genes remain with an unknown function (Cherry, 2015). In *Escherichia coli*, a high proportion of unannotated genes were found to be involved in microbial interactions, thus investigating biotic interactions may offer new biological insight into genotype to phenotype mapping (Pierce et al., 2021). In microbial ecology, identifying which genes are of importance for conserved or even specific microbial interactions is of interest as it contributes to understand their mechanism and their impact on species evolution. In that perspective, the recently introduced approach of performing co-cultures with collections of functional mutants (N’guyen et al., 2020; Pierce et al., 2021), which are available in an increasing number of species owing to technology such as transposon-insertion sequencing (Cain et al., 2020), is an interesting avenue to characterize microbial interactions.

In systems biology, network perturbations have been studied in a number of systems with often a trade-off between the number of interactions measured vs. the number of perturbations (Filteau et al., 2016). The same can be said about a rare example of network perturbation study in a model microbial community (Hsu et al., 2019). The authors investigated the interaction between three microorganisms in the presence of several antibiotics combinations and temperatures using a microfluidic approach. While they were able to test several perturbations, the number of interactions considered was limited. As higher-throughput interaction mapping approaches are developed, it is expected that our ability to test various contexts or perturbations will also increase, enabling hypothesis testing at the system level. For instance, emerging properties of networks such as modularity and connectivity, have been successfully studied in cell biology and shown to encode valuable biological insight (Gosak et al., 2018; Machado et al., 2021). In microbial systems, modularity in correlation network of binary interaction profiles could potentially reveal shared life history patterns that extend beyond phylogenetic relationships.

Dedicated resources such as databases [e.g., the *Saccharomyces* Genome Database (Cherry, 2015) and Biogrid (Oughtred et al., 2021)] and standardized functional annotations [the Gene Ontology initiative (Ashburner et al., 2000; Gene Ontology Consortium, 2020)] have been instrumental in enabling systems biology research by providing information about the various cellular components. Protein–protein interactions are a good example where results from several methods and studies have been combined to construct high-confidence networks (Machado et al., 2021). Their equivalents at the microbial community level are sorely needed, as existing databases of microbial interactions, phenotypes and functions are scarce and so far, mostly oriented toward gene annotation, e.g., (Chibucos et al., 2014; Urban et al., 2017). The consolidation of microbial interaction knowledge in a single database would be an invaluable resource. To enable quantitative comparative analysis across experimental systems and methods, Pacheco and Segrè (2019) proposed a framework to standardize interactions descriptions. However, the sheer quantity of data makes consolidation and curation a colossal task which could be aided by text-mining tools (Lim et al., 2016). Such initiatives are a step in the right direction to enable investigation of the architecture and emerging properties of microbial interaction networks.

Are conclusions derived from microbial interaction patterns applicable along different contextual scales? To begin addressing this question, we turned our focus on methods to map microbial relationships *in vitro*, and on different types of networks that can be constructed from experimental data. While we recognize that modeling microbial community behavior is the ultimate goal (Zaramela et al., 2021), we reason that the heuristic strategy of mapping microbial interactions at high throughput constitutes a necessary step to validate the emerging properties of microbial communities at the system level. Altogether, the studies included herein underline the need to pursue the development of shared resources, tools, and methods capable of mapping interactions experimentally at high throughput while accounting for the complex nature of microbial mechanisms and the diversity of microbial community functions.

## Author Contributions

GG, AN, and MF wrote the manuscript. GG and AN prepared the tables. MF prepared the figures. All authors contributed to the article and approved the submitted version.

## Funding

This work was funded by NSERC Discovery grants (funding reference number RGPIN-2017-04771) and FRQNT research support for new academics grant (funding reference number 270704) to MF.

## Conflict of Interest

The authors declare that the research was conducted in the absence of any commercial or financial relationships that could be construed as a potential conflict of interest.

## Publisher’s Note

All claims expressed in this article are solely those of the authors and do not necessarily represent those of their affiliated organizations, or those of the publisher, the editors and the reviewers. Any product that may be evaluated in this article, or claim that may be made by its manufacturer, is not guaranteed or endorsed by the publisher.
